# Morphometric analysis of the human common hepatic artery reveals a rich and accessible target for sympathetic liver denervation

**DOI:** 10.1038/s41598-022-05475-6

**Published:** 2022-01-26

**Authors:** Abraham Rami Tzafriri, Fernando Garcia-Polite, John Keating, Raffaele Melidone, Jennifer Knutson, Peter Markham, Elazer R. Edelman, Felix Mahfoud

**Affiliations:** 1 CBSET Inc., 500 Shire Way, Lexington, MA 02421 USA; 2 IMES, MIT, 77 Massachusetts Avenue, Cambridge, MA USA; 3 Cardiovascular Division, Brigham and Women’s Hospital, Harvard Medical School, Boston, MA USA; 4 Department of Internal Medicine III, Saarland University, Homburg/Saar, Germany

**Keywords:** Anatomy, Cardiology, Medical research, Cardiovascular diseases

## Abstract

This study quantified the distribution of nerves and adjacent anatomies surrounding human common hepatic artery (CHA) as guidance for catheter based denervation. CHA collected from cadaveric human donors (n = 20) were histologically evaluated and periarterial dimensions and distributions of nerves, lymph nodes, pancreas and blood vessels quantified by digital morphometry. Nerve abundance decreased significantly with distance from the aortic ostium (P < 0.0001) and was higher in the Superior/Inferior compared to the Anterior/Posterior quadrants (P = 0.014). In each locational group, nerves were absent from the artery wall, and starting 0.5–1.0 mm from the lumen exhibited a first order dependence on radial distance, fully defined by the median distance. Median subject-averaged nerve distance to the lumen was 2.75 mm, ranging from 2.1–3.1 mm in different arterial segments and quadrants and 2.0–3.5 mm in individuals. Inter-individual variance was high, with certain individuals exhibiting 50th and 75th nerve distances of, respectively, 3.5 and 6.5 mm The pancreas rarely approached within 4 mm of the lumen proximally and 2.5 mm more distally. The data indicate that the CHA is a rich and accessible target for sympathetic denervation regardless of sex and diabetes, with efficacy and safety most optimally balanced proximally.

## Introduction

Treatment of type 2 diabetes mellitus includes lifestyle modifications and medication regimens^1^ that require concerted efforts from both physicians and patients to be effective owing to high rates of noncompliance. Indeed, it has been estimated that non-adherence to antidiabetic drugs ranges from 53 to 65% and may be responsible for uncontrolled glucose levels in about 23% of patients^2^. Development of alternative approaches that are less prone to adherence issues could therefore offer a significant advancement in diabetes care. Given the association of activation of the sympathetic nervous system with altering insulin resistance^3^ and the development of type 2 diabetes mellitus^4^, catheter-based sympathetic denervation of the digestive system has been suggested as one such alternative^5^.

Physiological and anatomical studies^6–9^ both suggest the common hepatic artery (CHA) as a promising candidate for such interventions. Such a novel approach can build on the extensive experience with catheter-based renal denervation as therapy for hypertension^10^. Despite the recent string of successful clinical studies of renal denervation using second generation devices^11–13^, it is important to note that this was preceded by the failure of the first blinded randomized sham controlled study with the first generation single electrode radiofrequency device^14^. Postmortem histopathological analysis of human tissue samples demonstrated that radiofrequency ablation patterns failed to affect the majority of nerves in the renal artery periadventitia^15^. Moreover, concurrent preclinical animal and computational studies had predicted such procedural limitations based on a careful evaluation of the asymmetric periadventitial distributions of nerves^16^ and heat conducting and absorbing microanatomies such as muscles, lymph nodes, and adjacent blood vessels^17^.

The above evidence demonstrated the importance of defining the target microanatomy as a basis for optimizing the design of denervation catheters and treatment protocols^18,19^. The current study therefore set out to quantitatively determine the size and number distribution of nerves relative to the lumen of the human CHA, as a function of radial distance and orientation and the distance from the aortic ostium. As context for the potential efficacy and safety of CHA denervation, we also quantitatively defined the spatial distributions of periadventitial lymph nodes and adjacent vessels, as well as the pancreas.

## Results

Quantitative histomorphometric evaluation was performed on a total of 20 CHA to determine nerve type, dimensions, and distribution, as well as characterization of non-target anatomical sites (Table 1). Donors were 48–78 years old (65 ± 8.6) and distributed equally between male (n = 10) and females (n = 10). Per donor medical histories, eleven of the samples were designated diabetics, 9 non-diabetics. Diabetic and non-diabetic donors were similarly distributed in terms of sex, age, height, body mass index (BMI), hypertension, and renal disorders. Male donors were similar to females in age, diabetes, and BMI, but were 6 inches taller (P = 0.002).

**Table 1:** Patient characteristics.

	All (n = 20)	Diabetic (n = 11)	Nondiabetic (n = 9)	P value
Age, years	65	66	65	0.8682
Height, inches	67.2	68.5	65.7	0.4074
Sex (male/female)	10/10	6/5	4/5	ns
Race (White/African American)	19/1	10/1	9/0	ns
Overweight (BMI ≥ 25 kg/m^2^) (yes/no)	12/8	7/4	5/4	ns
Diabetes (yes/no)	11/9	11/0	0/9	–
Hypertension (yes/no)	8/12	4/7	4/5	ns
CKD	18/2	10/1	8/1	ns

Values are means or n. Categorical data were analyzed by using Fisher’s exact test (ns denotes P > 0.05). *BMI* body mass index, *CKD* chronic kidney disease.

**Figure 1:**
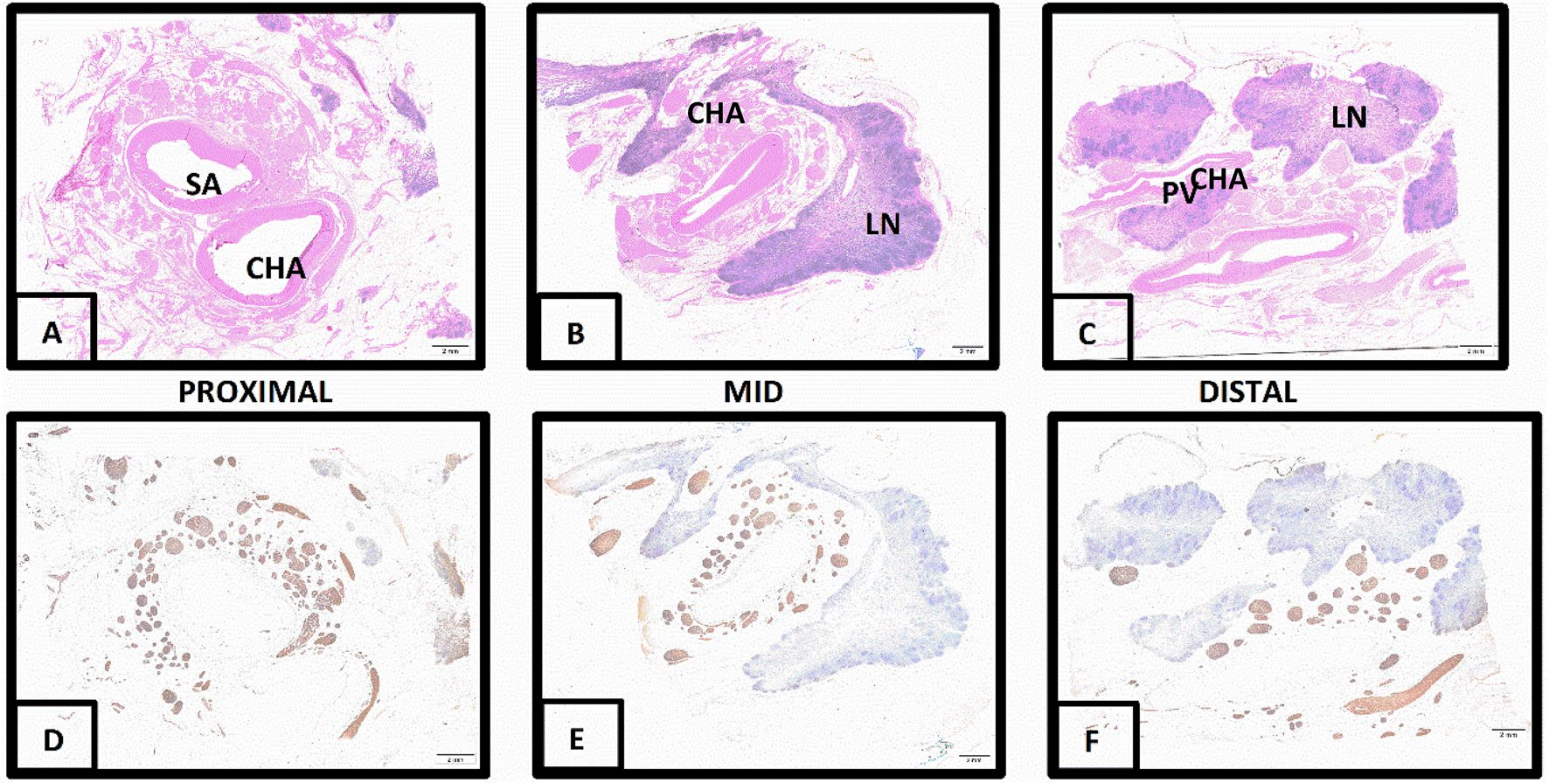
Longitudinal characterization of a representative common hepatic artery. Proximal, middle, and distal segments shown with H&E stain (**A**–**C**) and corresponding TH immunohistochemistry (**D**–**F**). H&E staining shows segments beginning just distal to splenic artery (SA) bifurcation. Prominent lymph nodes (LN) are present in (**B**) and (**C**). Portal vein (PV) branch is indicated. TH staining is strong and diffuse (brown color) within all nerve bundles. No ganglia are present. A 2 mm scale bar was used for all 6 panels.

Tissue sections across the length of the CHA exhibited abundant nerves within the loose adventitial/periarterial adipose and fibrovascular tissue that were often surrounded by lymph nodes, pancreas and vascular structures, including comparably sized portal veins and smaller arterial and venous branches (Fig. 1). Nerves were non-myelinated, arranged in thin-walled, irregular fascicles. No associated ganglionic structures were present. There was strong diffuse Tyrosine hydroxylase (TH) staining of periarterial nerves confirming their predominantly sympathetic nature. Staining of representative CHA sections from each sample for calcitonin gene related peptide (CGRP) was either entirely absent or negligible within nerve fibers (Supplemental Fig. 2) and consistent in such cases with supportive cells rather than axonal fibers. Overall nerve fiber positivity for CGRP was estimated at < 5%, and afferent fibers were therefore considered a negligible fraction within periarterial nerves.

A total of 11,747 nerves were identified in 116 CHA sections with 81.5% (9574) of these determined to be CHA associated, e.g. not associated adjacent blood vessels, or even spatially shielded from possible CHA treatment by adjacent blood vessels and lymph nodes. Morphometric parameters for CHA-associated nerves are shown in Table 2 along with a comparison of sex and diabetic status. As evident in the representative TH stains (Fig. 1) CHA associated nerves were abundant (81.6 ± 17.6/section) and frequently large (0.35 ± 0.05 mm). Nerve size, abundance (total and per section) and distance from the lumen were all uncorrelated with either age or BMI, but nerve abundance per section and total nerve area per section both correlated significantly with donor height (respectively r = 0.692, p = 0.001 and r = 0.584, p = 0.007). However, while nerve size was independent of sex, diabetics exhibited 28% larger nerves versus nondiabetics (0.18 vs. 0.14 mm^2^, P = 0.028). Moreover, females exhibited a 9% lower nerve abundance per section (77.6 vs. 85.61/section, P = 0.036) and 22% less nerve area/section (11.7 vs. 15.0 mm^2^/section, P = 0.032). Donor averaged median (*r*_*50%*_) and 75th percentile (*r*_*75%*_) nerve distances from the lumen were, respectively, 2.75 and 4.5 mm. However, variance was high, with certain donors exhibiting more spatially extended nerve distributions with *r*_*50%*_ = *3.5* mm and *r*_*75%*_ = 6.5 mm Supplemental Fig. 3). Notably median nerve distance from the CHA lumen was 0.5 mm larger for males versus females, though this trend did not attain statistical significance (P = 0.067).

**Table 2:** CHA associated nerve parameters classified by sex and diabetic status.

	Number of CHA samples (number of sections)	Nerves/section	Total nerve area/section (mm^2^)	Nerve distance to lumen (mm)	Nerve area (mm^2^)	Nerve diameter (mm)
Total	20 (116)	81.6 ± 17.6	13.3 ± 3.5	3.6 ± 0.6	0.16 ± 0.15	0.35 ± 0.05
Male	10 (62)	85.6 ± 12.8	15.0 ± 8.1	3.7 ± 0.6	0.17 ± 0.07	0.36 ± 0.06
Female	10 (54)	77.6 ± 28.5	11.7 ± 4.2	3.5 ± 0.5	0.15 ± 0.04	0.35 ± 0.04
P value male vs. female	NA	0.04	0.032	0.40	0.75	0.61
Nondiabetic	9 (56)	87.4 ± 25.9	12.3 ± 5.3	3.5 ± 0.6	0.14 ± 0.04	0.33 ± 0.04
Diabetic	11 (60)	76.8 ± 17.9	14.2 ± 7.6	3.6 ± 0.5	0.18 ± 0.06	0.37 ± 0.05
P value normal vs. diabetic	NA	0.26	0.40	0.65	0.03	0.04

Values are n or mean ± SD. Student *t* test with unequal variances or Mann–Whitney test as appropriate.

**Figure 2:**
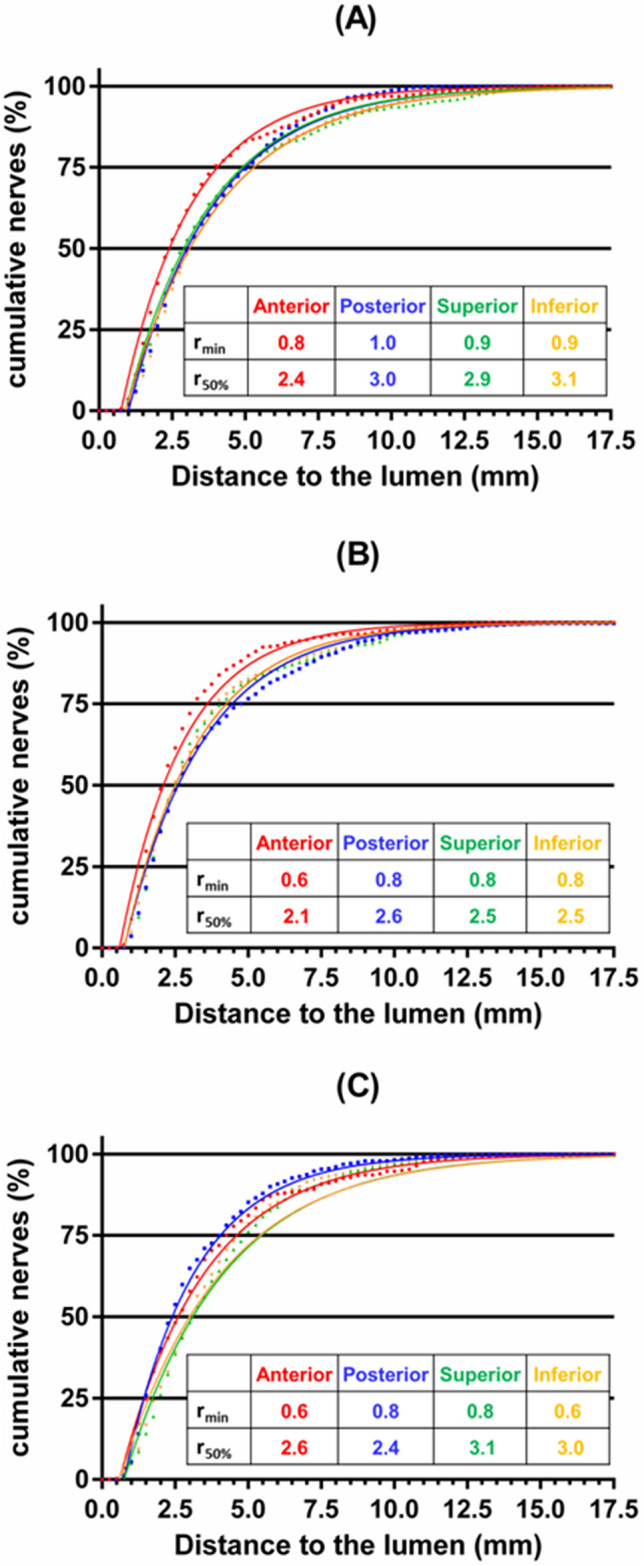
Radial nerve distribution varies with orientation and distance from aortic ostium. Composite radial nerve distributions are plotted for each of the four quadrants of the (**A**) proximal, (**B**) middle and (**C**) distal segments of the CHA. Composite data sets are depicted in 0.25 mm bins (dots) and contrasted with Eq. (1) (solid lines) using the tabulated minimal (*r*_*min*_) and median (*r*_*50%*_) nerve distances directly obtained from the data.

Overall, cumulative nerve distribution was negligible up to a minimal distance *r*_*min*_ = 0.5–1.0 mm and thereafter rose exponentially (Fig. 2A–C). Consequently, the distribution beyond the CHA wall can largely be characterized by the median nerve distance from the lumen, *r*_*50%*_ Median nerve distance varied as nerves coursed away from the ostium, and within each segment also with quadrant. The proximal segment exhibited the maximal median nerve distance (*r*_*50%*_ = 3.0 mm), with the anterior quadrant exhibiting the nadir (*r*_*50%*_ = 2.4 mm). As nerves continue to course into the middle segment they distribute most closely to the lumen (*r*_*50%*_ = 2.5 mm), once more with a nadir in the anterior quadrant (*r*_*50%*_ = 2.1 mm). In the distal segment, nerve distance from the lumen rises once more (*r*_*50%*_ = 2.75 mm) with the nadir in the posterior quadrant (*r*_*50%*_ = 2.4 mm), though nerves in the anterior quadrant are also substantially closer to the lumen (*r*_*50%*_ = 2.6 mm) compared to the superior and inferior quadrants. Overall, nerves were closer to the CHA lumen in the Anterior quadrant compared to the Inferior/Posterior/Superior quadrants (P = 0.004).

**Figure 3:**
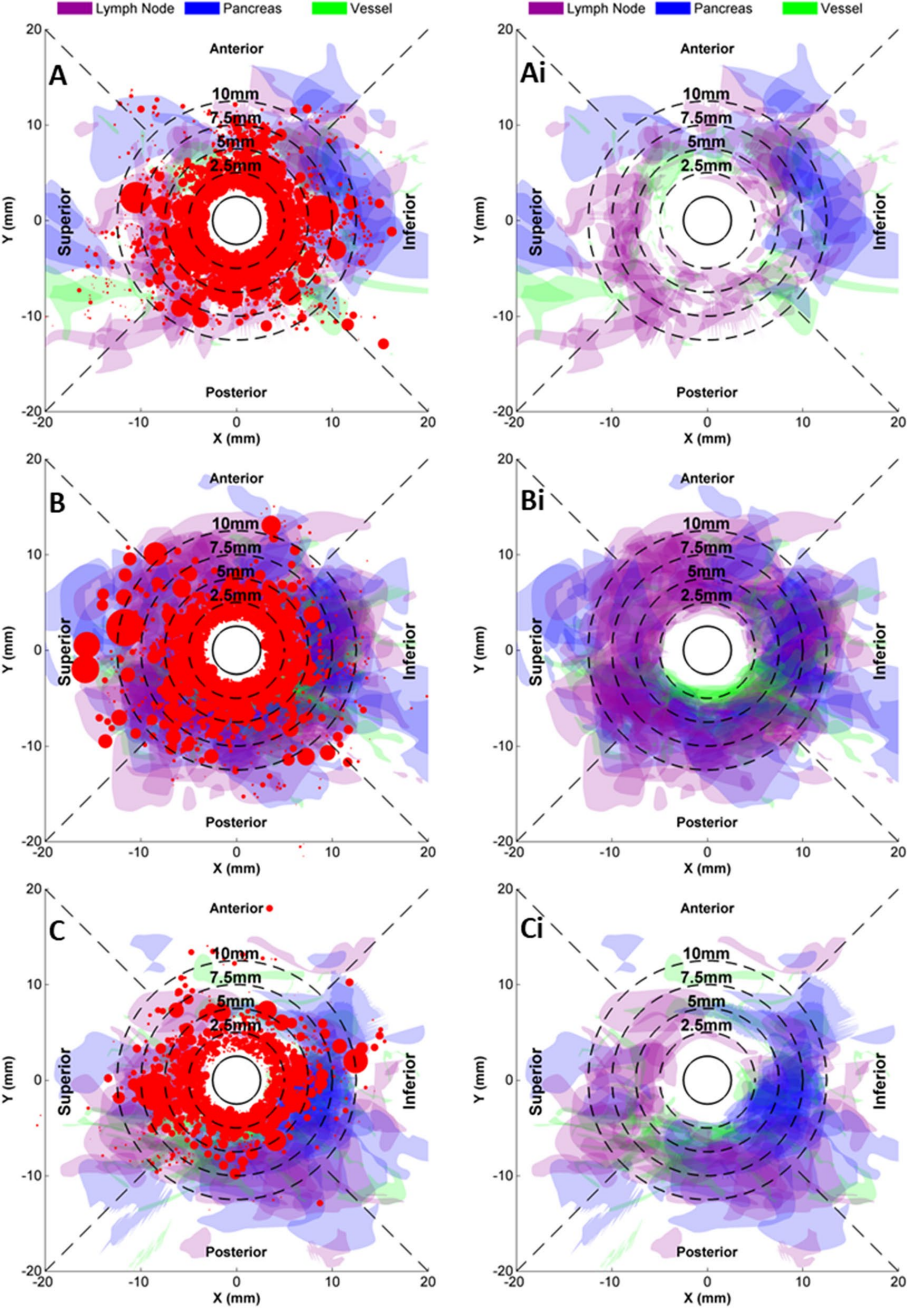
Periarterial CHA microanatomy varies with distance from aortic ostium and with orientation. Standardized composite microanatomy maps of the CHA classified by longitudinal location, (**A**,**Ai**) proximal, (**B**,**Bi**) middle and (**C**,**Bi**) distal, and depicted for each of the four quadrants. Three sequential views are presented per segment, from proximal to distal (arrow) with each depicting areas associated with nerves (red) lymph nodes (purple), pancreas (blue) and adjacent blood vessels (green). (**Ai**–**Ci**) Depict the surrounding anatomies without the obscuring nerves. Greater color intensities imply greater local lymph node, pancreas, or vessel composite densities.

Micro-anatomical dissection and image analysis revealed the segment and circumferential dependence of nerve abundance and distribution (Fig. 3A–C). As nerves coursed along the CHA distally from the aorta, their abundance and total area per section declined 1.9-fold (P < 0.001) and 1.6-fold (P = 0.018) respectively between proximal and middle segments and a further 25% (P = 0.087) and 29% (P = 0.19) respectively between the middle and distal groups. On the contrary, the size of individual nerves increased by 29% between the proximal and distal ends of the CHA (proximal: 0.14 ± 0.05 mm^2^, distal: 0.18 ± 0.05 mm^2^, P = 0.094). In all three segments, polar distribution maps revealed a dense and relatively uniform nerve abundance between the tunica media and up to a distance of 2.5 mm from the lumen (2.5 mm circle in Fig. 3A–C), though the anterior quadrant of the proximal segment exhibited a slightly lower nerve density. Between 2.5–5.0 mm from the lumen, nerve distribution remained uniformly dense at all angles for the proximal and middle segments (Fig. 3A,B), but not for the distal segments which exhibited nerve free areas in the anterior and posterior quadrants (Fig. 3C). Beyond 5.0 mm from the lumen, nerve distribution is more sporadic and tends to be dominated by larger nerves, especially in the Superior and Inferior quadrants. Overall, nerve abundance was equivalent in the Superior and Inferior quadrants and significantly higher than the Anterior and Posterior quadrants together (P = 0.014).

CHA adjacent anatomic structures were also mapped and quantified as a function of their orientation and distances from the lumen and the aorta to examine how these might affect CHA associated nerve distribution and as context for therapy design. Lymph nodes, adjacent vessels and pancreas were largely absent up to radial distance of 2.0–2.5 mm from the lumen along the length of the CHA (Fig. 3Ai–Ci). Further than 2.5 mm from the lumen, lymph node, pancreas and vessel distribution all exhibited defined quadrant and segment dependencies. Lymph nodes were abundant at all orientations of the proximal and middle segments, but as one progresses more distally their abundance decreased in the anterior quadrant. The incidence of pancreas presence in tissue sections was even more contextual, with especially low prevalence in the posterior quadrant of the proximal segment and anterior quadrant of the distal segment. Overall, pancreas area per section was highest in the inferior quadrant and lowest in the anterior quadrant. Adjacent vessel distribution appeared to be the most localized, with high incidence 2.0–5.0 mm from the lumen observed in the anterior quadrant of the proximal segment and the posterior quadrants of the middle and distal segments.

As ablative therapies must balance efficacious nerve ablation with preservation of non-target vital structures, we quantified their minimal distances to the CHA lumen for each of the histological sections. Minimal distances from the lumen varied considerably between and within CHA samples, with longitudinal position and quadrant. The closest measured distances to the lumen in any of the sections were 0.8 mm for lymph nodes, 1.2 mm for the pancreas and 0.5 mm for the vessels. The farthest minimal measured distances to the lumen in any of the sections were 10.7 mm for lymph nodes, 12.2 mm for the pancreas and 13.6 mm for the vessels. Median minimal distances from the lumen for composite data set (Supplemental Fig. 4) was largest for the pancreas (2.8 mm), followed by adjacent vessels (1.8 mm) and lymph nodes (1.6 mm)—all equal to or smaller than the median nerve distance from the CHA lumen (2.75 mm). The gender and diabetes dependences of minimal microanatomy distances from the CHA lumen were also tested (Supplemental Fig. 4), and no statistically significant differences were found, though the median minimal distances from the lumen of the pancreas and adjacent vessels trended 50% higher for males versus females.

Minimal non-target microanatomy distances from the CHA lumen were also quantified as a function of segment (Supplemental Fig. 4A–C) and orientation (Supplemental Fig. 5Ai–Ci). Minimal distances of all three non-target structures were largely independent of orientation and in the case of lymph nodes, also independent of segment (Supplemental Fig. 5A). Minimal pancreas and adjacent vessel distances from the lumen both exhibited a remarkably similar statistically significant dependence on orientation, with both peaking in the proximal segment, declining two-fold in the middle segment and then increasing in the distal segment to approximately 75% of the proximal value.

## Discussion

**Figure 4:**
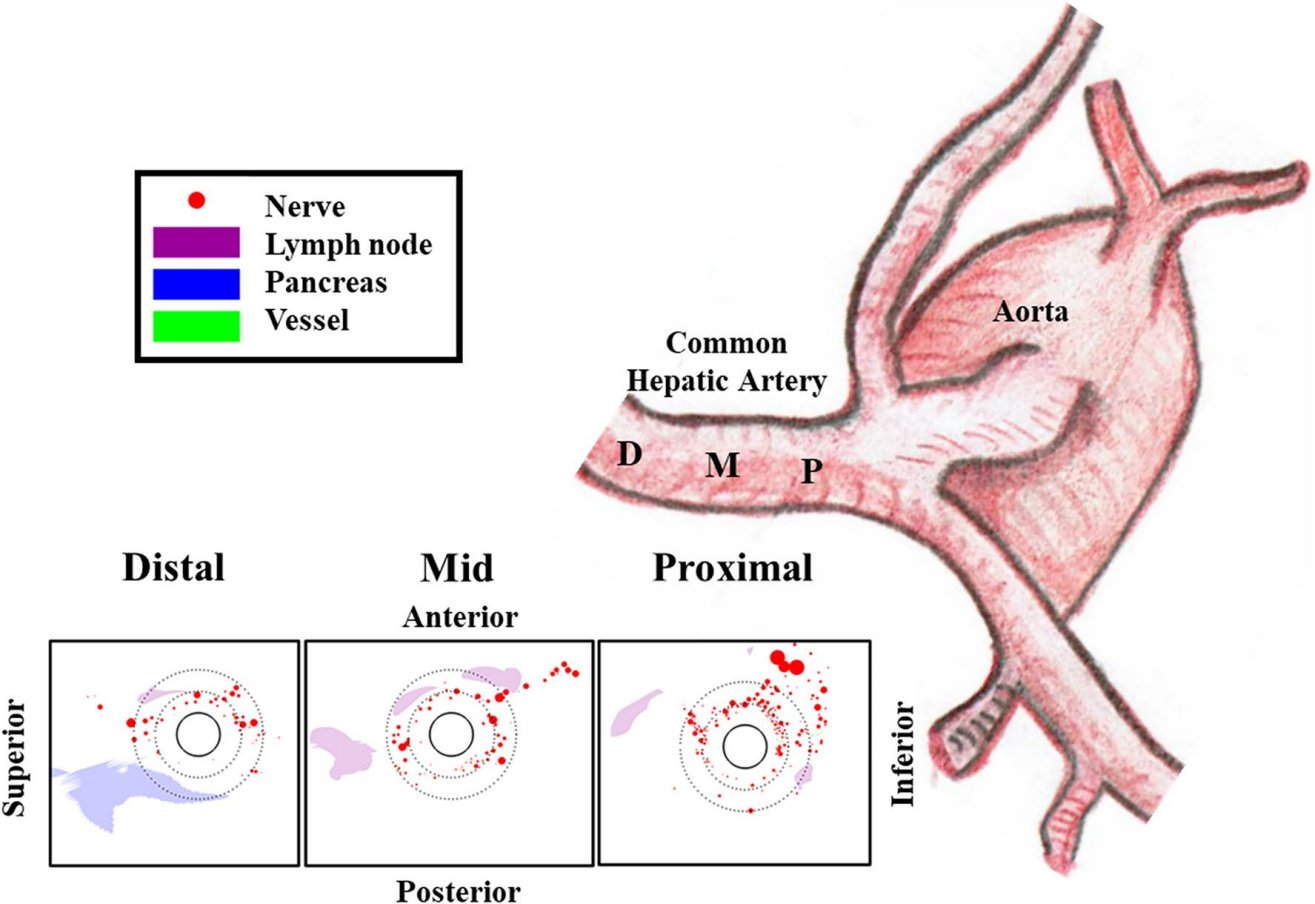
Central illustration: asymmetric periarterial innervation and anatomies. Histological sections reveal a complex periarterial anatomy rich not only in target nerves, but also in adjacent lymph nodes, vessels and pancreas that should be spared from ablation. Nerve abundance decreases with distance from the aorta whereas minimal pancreas distance from the lumen is minimal closest to the aortic ostium.

Percutaneous endovascular denervation of the liver via the CHA, which may alter overactive sympathetic signaling to the pancreas and liver, is under clinical investigation as a novel diabetes treatment^20^. The efficacy and safety of the therapy depends on achieving targeted ablation of a sizable fraction of sympathetic nerves associated with the CHA while sparing adjacent vital tissue, such as the pancreas, adjacent lymph nodes and blood vessels (including their own nerves). In support of these goals, the current study evaluated the sympathetic nature of nerves surrounding the CHA of human arteries as well as their detailed spatial distribution relative to the arterial lumen, the aortic ostium, the pancreas, lymph nodes and adjacent blood vessels (Fig. 4).

Peri-CHA nerves were predominantly postganglionic sympathetic efferent with strong diffuse TH staining (Fig. 1) confirming their sympathetic nature, and a virtual absence of CGRP staining identifying them as being mostly efferent. Moreover, of the total identified 11,747 nerves in the evaluated samples, 81.5% (9574) were determined to be anatomically associated with the CHA (or at least not anatomically associated with a different structure), and to not be spatially shielded from possible CHA treatment by other structures (e.g., blood vessels, lymph nodes). Compared to reported values on peri-renal nerves^21^, CHA associated nerves were three-fold larger (0.35 vs. 0.12 mm) and two-fold more abundant per tissue section (81.6 vs. 37.5 nerves/section). Nerve size was independent of donor age, BMI and sex, but was 13% larger for diabetics versus nondiabetics (0.37 vs. 0.33 mm, P = 0.036). While nerve abundance per section was also independent of age and BMI, it was 10% higher in males compared to females (P = 0.036) but independent of diabetes. Nerve abundance per section also significantly correlated with height, though this appears to be an artifact of its sex dependence as male donors were significantly taller than female donors. To further characterize the accessibility of CHA associated nerves to catheter-based liver denervation, we quantified their distance from the lumen across the length and arc of this artery. In animal studies, ≥ 50% ablation of renal sympathetic nerves has been shown to associate with a significant decline in norepinephrine, with higher percentages of nerve ablation correlating with more significant declines in this marker for sympathetic activity. Median and 75th percentile nerve distance to the CHA lumen were respectively 2.75 and 4.5 mm, 10% larger than reported estimates for peri-renal nerves in human samples^21^. Median nerve distance from the CHA lumen was independent of diabetes, but did trend 19% higher in males compared to females (3.025 vs. 2.550, P = 0.067). Cumulative radial nerve distribution exhibited a first order rise with distance from the artery wall with a median nerve distance that varied between 2.1–3.1 mm along the length and circumference of the CHA (Fig. 2). However, inter-individual variance was high, with median nerve distance ranging from 2.0–3.5 mm and one donor exhibiting a 75th nerve distance reaching 6.5 mm (Supplemental Fig. 3). Encouragingly, a recent preclinical study has demonstrated that even such distant nerves are within reach of specific technologies^5^.

As nerve accessibility to intravascular ablation did not vary with location along the length and circumference of the CHA, the attractiveness of treating a particular location depends more on local nerve abundance and the concern of targeting adjacent organs, particularly the pancreas. Thus, it is worth noting that nerve abundance depended significantly on location (Fig. 3), decreasing significantly with distance from the aortic ostium (P < 0.001) and exhibiting significantly higher levels in the superior/inferior quadrants compared to the anterior/posterior (P = 0.014). However, the latter angular dependence reflects an overabundance of nerves > 5 mm from the lumen in the superior/inferior quadrants. In general, 20% of nerves are located beyond 5.0 mm from lumen, and while of larger size their distribution tends to be more sporadic.

Median minimal distances from the lumen for the composite data set were largest for the pancreas (2.8 mm), followed by adjacent vessels (1.8 mm) and lymph nodes (1.6 mm)—all equal to or smaller than the median nerve distance from the CHA lumen. However, in certain locations, these off-target structures are much closer to the potential luminal treatment site. This can result in some shielding of approximately 18.5% of nerves from ablation by lymph nodes or adjacent blood vessels. Of greater concern, the minimal pancreas distance from the lumen was as low as 1.2 mm, well within the range of ablation catheters, highlighting the importance of anticipating locations of safety concern for novel therapies. This may be more of a concern for females as the median minimal distances from the lumen of the pancreas and adjacent vessels trended, respectively 1.0 mm (P = 0.168) and 1.3 mm (P = 0.075) higher for males versus females, though statistical significance was not achieved. This concern is somewhat tempered by the trend for 0.5 mm lower median nerve to lumen distance in females compared to males (P = 0.067). No similar dependences on diabetes were observed.

One can only speculate as to the reason for greater separation of the pancreas, lymph nodes and blood vessels from the CHA. Given that male donors were significantly taller than females, their torsos are longer allowing for greater organ separation. However, minimal pancreas to liver distance did not correlate with donor height, leaving the intriguing possibility that the greater inter-organ distances in males arise from adipose tissue being more abundantly distributed around male livers. Unfortunately, we did not anticipate and record such potential differences.

## Limitations

Despite pressure-perfusion fixation of samples, histological samples were not perfectly circular, thereby limiting the ability to define the centroid and angles with consistent accurately. While this lack of perfect circularity limits the accuracy of two-dimensional microanatomical maps, it should only negligibly impact the quadrant level findings. However, based on the literature^21,22^ and our own experience, histological processing is known to cause tissue shrinkage on the order of 20–30%. Thus, the reported histological-based distances may under-estimate the corresponding distances in live patients but remain useful in comparing interpatient differences in our study, and to published data on human cadaver renal arteries that were similarly processed. Although the investigated sample size of tissue sections and nerves was large, the number of autopsy cases was limited (n = 20) and we cannot rule out that the observed trends for greater nerve, pancreas and adjacent vessel distances from the CHA lumen in males versus females did not achieve statistical significance is due to sample size. These trends which raise the intriguing prospect that ablation therapies be tailored to patient sex and or height, are worth further examination in larger data sets that better control for patient height.

## Conclusions

The reported human cadaver analysis provides useful guidance on both average anatomical trends and patient variability. Per our data, the pancreas is most distant from the lumen in the proximal segment, rarely approaching within 4 mm from the lumen. In the middle and distal segments, the pancreas rarely approaches the lumen to within, respectively, 2.5 and 3.0 mm. Ablation therapy protocols can take advantage of this information by titrating therapy based on distance from the aorta. Taken together with the overabundance of nerves in the proximal segment, this makes the near ostium regions particularly attractive for catheter-based liver denervation.

## Methods

### Ethics statement

Study results are presented for postmortem human anatomical tissues sourced from AATB (American Association of Tissue Banks) accredited tissue banks (ensuring donation by legal next of kin for medical science research purposes).

### Samples, blocking and immunohistological staining

CHA with attached aorta, celiac artery, and splenic artery were collected from 20 human anatomical samples supplied by AATB accredited tissue bank (Science Care, Phoenix, AZ). To ensure morphological preservation under histological processing, the search criteria for these donors included the requirement of early refrigeration after demise and all the samples were supplied ≤ 72 h postmortem. Donor reports included information on sex, race, height, body mass index (BMI) and diabetes mellitus, hypertension, and renal disease determined from the medical chart of the donor and/or family interview. Samples were perfusion fixed ex vivo using a syringe to inject 10% neutral buffered formalin followed by incubation in the same solution for two weeks. Fixed specimens were then debulked and dissected to isolate the CHA while leaving sufficient periarterial tissue to allow thorough histologic evaluation of the intact periarterial microanatomy. No separation of the surrounding tissue was detected during or after dissection. Anatomic orientation was maintained via use of trimming notes, tissue ink (e.g., color code for quadrant locations) and photographic documentation. The entire CHA was sectioned in consecutive fashion into 3–5 mm blocks. Tissues were processed and paraffin embedded, slides were obtained from each block and stained with Hematoxylin and Eosin (H&E) for morphology assessment and immunohistochemical stains for nerve identification. Tyrosine hydroxylase (TH), part of the norepinephrine synthetic pathway, was used as a specific immunohistochemical marker for efferent sympathetic nerve fibers. Calcitonin Gene Related Peptide (CGRP) was used as a marker for afferent (i.e., sensory) nerve fibers^21^. Immunostaining was achieved using rabbit anti-tyrosine hydroxylase (1:700, AB112; Abcam, Cambridge, United Kingdom) and mouse anti-CGRP (1:4000, Ab81887; Abcam, Cambridge, United Kingdom) primary antibodies with species-specific secondary antibodies.

### Micro-anatomical mapping

Slides were converted into calibrated digital images by use of an Olympus vs120 slide scanner using the 4 × objective. Scanned H&E slides were used for microanatomical mapping under pathologist guidance. CHA, nerves, lymph nodes, pancreas and adjacent vessels were identified on each image by the study pathologist. Using Fiji image analysis platform, all structures and the lumen of the CHA were outlined and saved as regions of interest (Supplemental Fig. 1A). As many of the observed nerves were distant from the target artery, the pathologists categorized each nerve as either CHA associated or not. CHA associated nerves were those that satisfied two complimentary criteria rendering them most likely accessible to transarterial ablations: (A) nerves were anatomically associated with the CHA (or at least not anatomically associated with a different structure), and (B) not spatially shielded from possible CHA treatment by other structures (e.g., blood vessels, lymph nodes). Nerve categorization was implemented via sequential review of slides per donor, and when in doubt adjacent prior and succeeding slides were evaluated for confirmation. One pathologist performed the preliminary assessment and categorization of all the slides (coauthor R.M.) and the second pathologist (coauthor J.K.) reviewed and confirmed the categorization. In the rare instances of discrepancies in categorization, these were discussed, and the final categorization reflects the consensus that was reached after further evaluation.

A dedicated Fiji script was implemented on the entire image data set. For each image, after evaluating the CHA centroid the image was rotated as needed to maintain a consistent orientation for vascular quadrants. For each CHA associated nerve, its area, orientation relative to the centroid and its distance along the lumen-centroid direction were recorded. For adjacent anatomies, minimal distance was calculated as the smallest distance to the lumen of any of the pixels defining the shape of the structure. Standardized two dimensional microanatomical maps of each image were generated using MATLAB Version R2020a (MathWorks, Natick, MA) after shifting individual radial positions by a representative constant lumen radius of 2.5 mm in an idealized circular vessel cross-section (Supplemental Fig. 1B), with nerve areas proportional to measurements (not to scale) and quadrant locations denoted (Anterior, Posterior, Superior and Inferior). Peri-arterial anatomies were represented as semi-transparent colored patches representing the normalized presence of pancreas, lymph nodes and vessels around the circular CHA. The use of standardized maps facilitated the aggregation of morphometric data from multiple slides to obtain patient level and population level composite maps in each of the longitudinal groups.

### Data analysis

Data reported are expressed as mean ± standard deviation (SD) and in some cases also as medians. Individual metrics per nerve were used to calculate average metrics per patient and per group (including sex). For each CHA, the number of sections was divided proportionally into Proximal, Middle and Distal segment groups relative to the CHA-splenic branching of the celiac artery. When no proportional subdivision was possible, the number of Proximal and Distal sections was kept constant and the rest of the sections were assigned to the Middle group.

All statistical analyses were performed using Graphpad Prism 6.0 (GraphPad Software, La Jolla CA). Correlations between donor age, BMI, and morphometric data were quantified using Spearman correlation coefficients. Categorical data were analyzed using Fisher’s exact test. For pairwise statistical comparisons of continuous variables, data were first assessed for normality using a Shapiro–Wilk test. If normality was met, a two-sided Student *t* tests with unequal variances (Welch’s correction) was conducted; otherwise, a Mann–Whitney rank sum test was performed. Differences were considered statistically significant with P < 0.05.

Radial cumulative nerve distributions were constructed by binning nerves in 0.25 mm radial intervals. These were contrasted with a shifted exponential rise (Eq. 1)^19^

1 $$Y = 100\left( {1 - e^{{\frac{{ - ln2 \cdot (r - r_{min} )}}{{r_{50\% } - r_{min} }}}} } \right),$$

where *x* is the distance from the lumen, and *r*_*min*_ and *r*_*50%*_ are respectively, the measured minimal and median nerve distances from the lumen. For a more complete representation of the nerve distribution we also tabulated *r*_*75%*_*,* the 75th percentile nerve distance from the CHA lumen.

## Supplementary Information

Supplementary Figures.
