# Correction: Morphometric analysis of the human common hepatic artery reveals a rich and accessible target for sympathetic liver denervation

**DOI:** 10.1038/s41598-025-12692-2

**Published:** 2025-08-06

**Authors:** Abraham Rami Tzafriri, Fernando Garcia-Polite, John Keating, Raffaele Melidone, Jennifer Knutson, Peter Markham, Elazer R. Edelman, Felix Mahfoud

**Affiliations:** 1 CBSET Inc., 500 Shire Way, Lexington, MA 02421 USA; 2 IMES, MIT, 77 Massachusetts Avenue, Cambridge, MA USA; 3 Cardiovascular Division, Brigham and Women’s Hospital, Harvard Medical School, Boston, MA USA; 4 Department of Internal Medicine III, Saarland University, Homburg/Saar, Germany

Correction to: *Scientific Reports* 10.1038/s41598-022-05475-6, published online 26 January 2022

The original version of this Article contained an error in Figure 4, where the annotation letters ‘M’ and ‘P’ did not appear closer to the letter ‘D’ and in the same arterial trunk. The original Figure 4 and accompanying legend appear below.

**Fig. 4:**
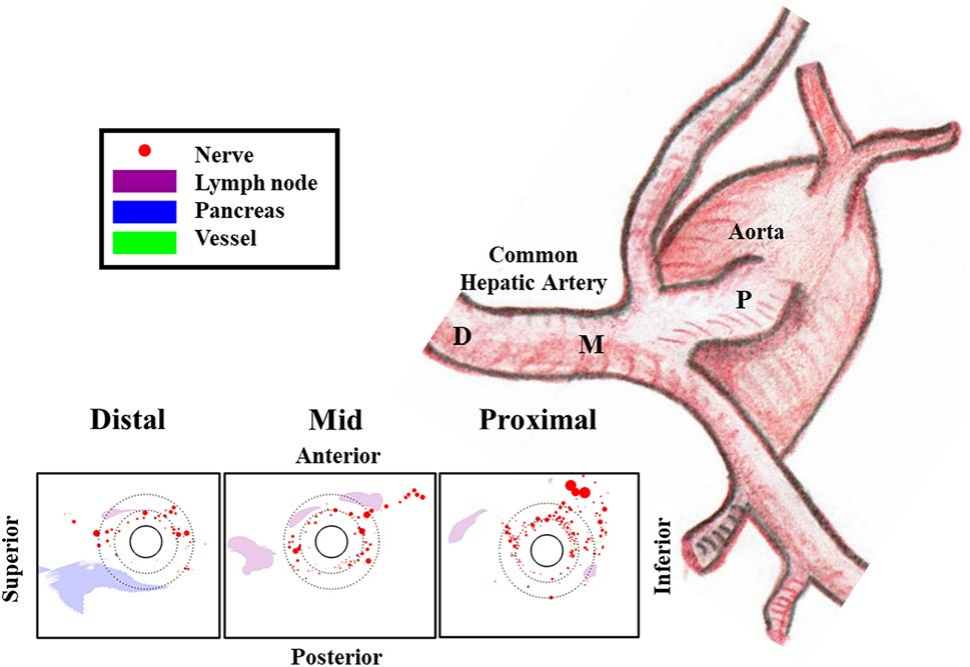
Central illustration: asymmetric periarterial innervation and anatomies. Histological sections reveal a complex periarterial anatomy rich not only in target nerves, but also in adjacent lymph nodes, vessels and pancreas that should be spared from ablation. Nerve abundance decreases with distance from the aorta whereas minimal pancreas distance from the lumen is minimal closest to the aortic ostium.

The original Article has been corrected.

