# Supplementary material for: Morphometric analysis of the human common hepatic artery reveals a rich and accessible target for sympathetic liver denervation
Source: Sci Rep. 2022 Jan 26;12:1413. doi: 10.1038/s41598-022-05475-6 (PMC8792043; doi:10.1038/s41598-022-05475-6)
Supplement: Supplementary file 1 — Supplementary Figures. [file 41598_2022_5475_MOESM1_ESM.docx]

SUPPLEMENTAL


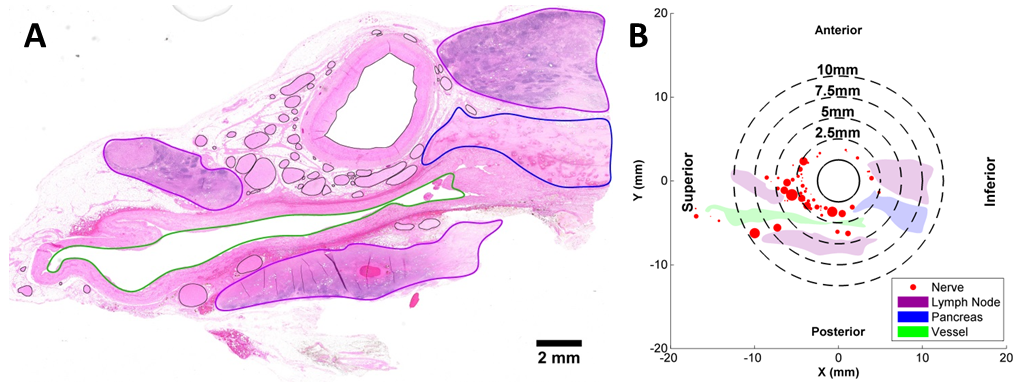


Panc

PV

LN

LN

LN

**CHA**

Supplemental Figure 1 Nerve and peri-arterial anatomy mapping process illustrated on a distal section from one of the donor samples. (A) Representative image with overlay of nerves (red dots) and CHA outlines (black), lymph nodes (purple), pancreas (blue) and adjacent vessel (green). (B) Corresponding standardized distribution map. Depicted nerve sizes are proportional to their measured values but not to scale.


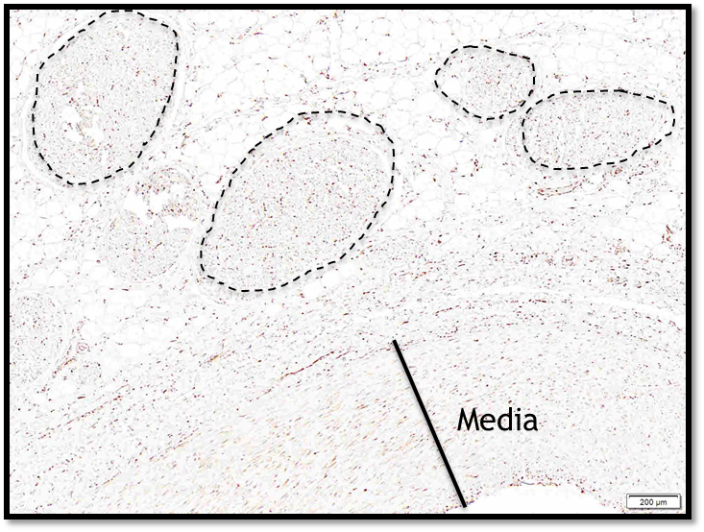


**Supplemental Figure 2** View from periphery of CHA shows that nerve fiber CGRP staining is absent in periarterial nerves (circled). Nerve staining includes few non-neuronal cells and is comparable to that of adjacent arterial adventitia and media (indicated by solid line).


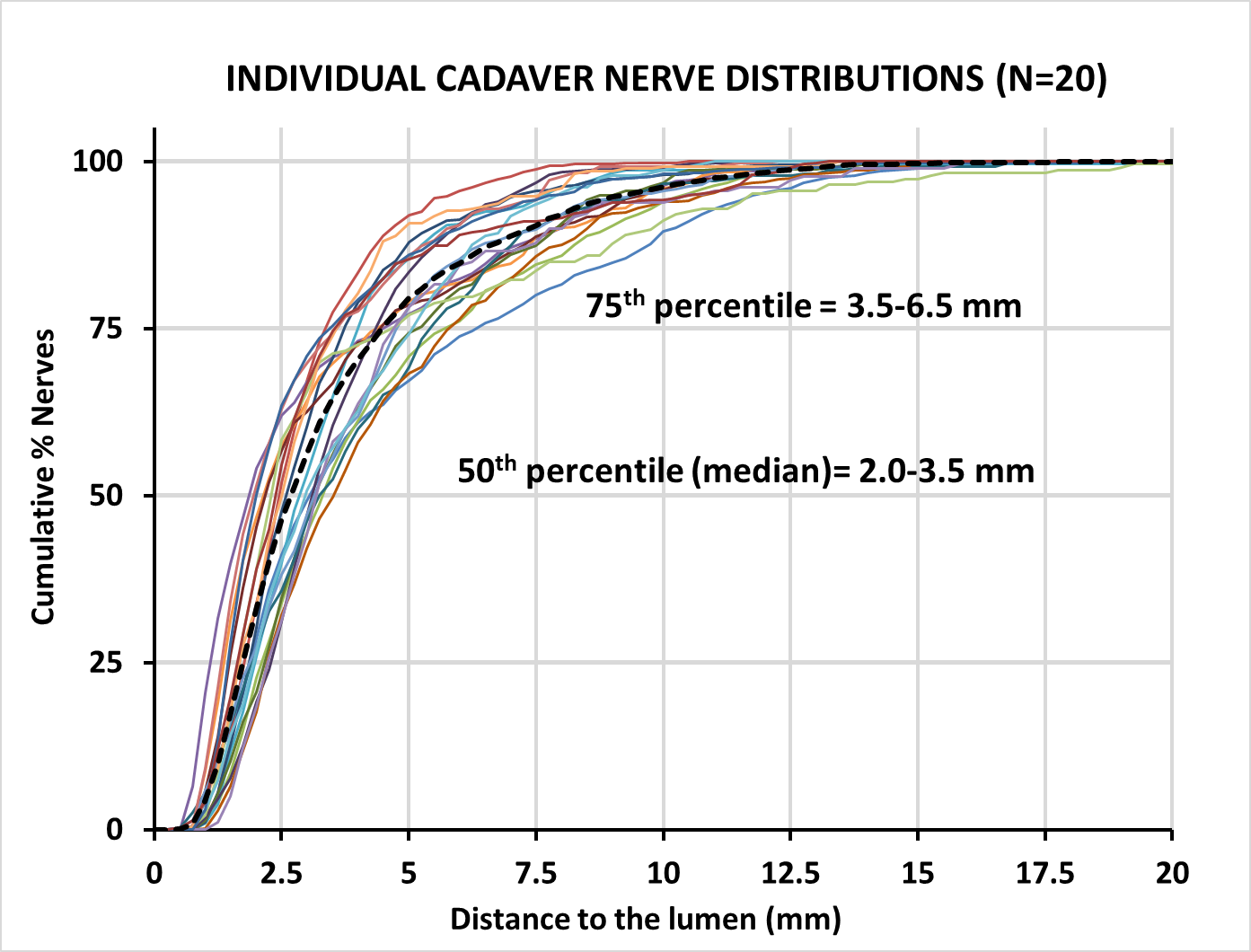


**Supplemental Figure 3** Subject specific nerve distribution relative to the CHA lumen exhibits a high degree of variance. Individual distributions are depicted by colored lines and the subject averaged distribution by black dashes.


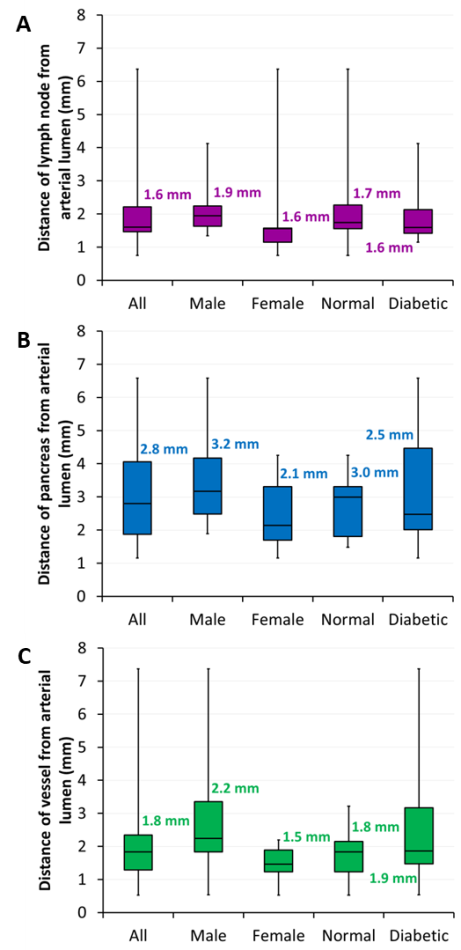


Supplemental Figure 4. Sex and diabetes dependence of the minimal distances of non-target microanatomies from the lumen. Box plots of minimal distance distributions of lymph nodes (A), pancreas (B) and vessels (C) for all patient samples analyzed grouped by sex and disease state. Asterisks denote statistically significant comparisons.


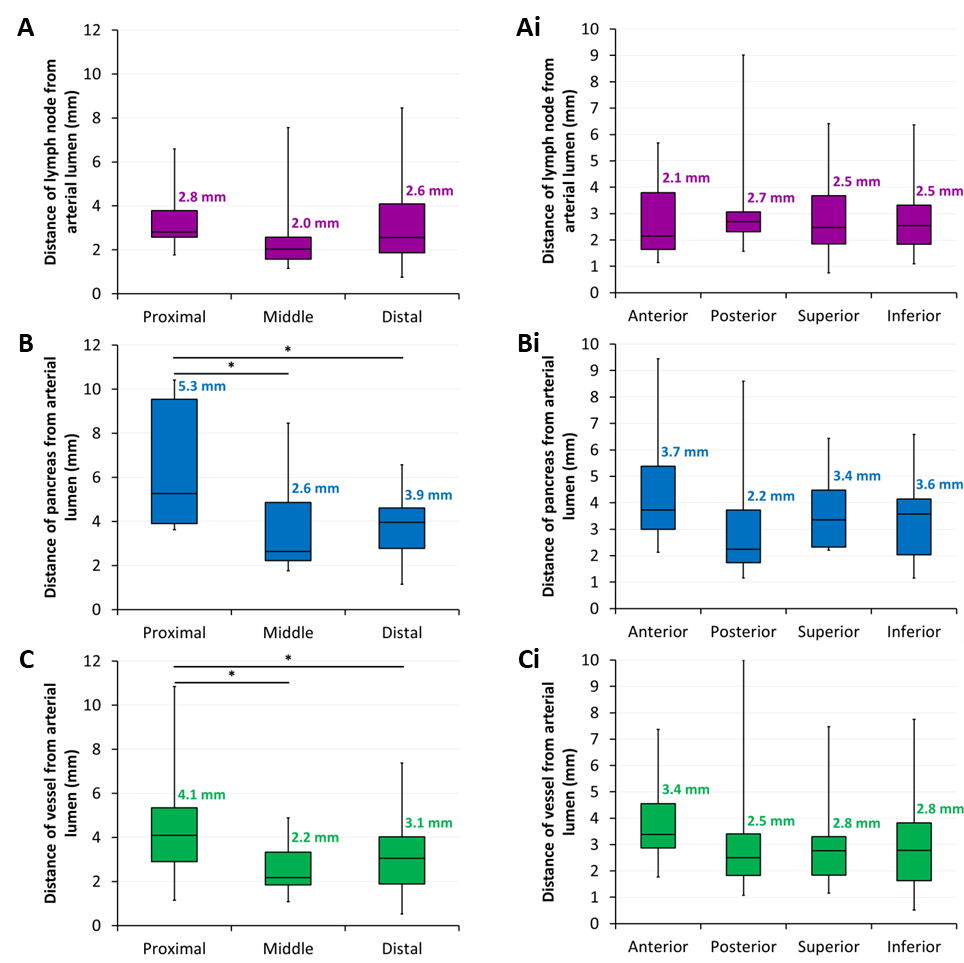


Supplemental Figure 5. Minimal distances of non-target micro-anatomies from the CHA lumen vary with distance from aorta (A-C) and with quadrant (Ai-Ci). (A, Ai) Lymph nodes (A), (B, Bi) pancreas and (C, Ci) vessels. Bar edges: quartiles 1 and 3, Bar intersection: median, indicated in the figure). Asterisks denote statistically significant comparisons.
